# A convenient method for determining the concentration of hydrogen in water: use of methylene blue with colloidal platinum

**DOI:** 10.1186/2045-9912-2-1

**Published:** 2012-01-24

**Authors:** Tomoki Seo, Ryosuke Kurokawa, Bunpei Sato

**Affiliations:** 1 MiZ Company, 16-5, Zengyo 1-chome, Fujisawa-shi, Kanagawa-ken 251-0871, JP

**Keywords:** Hydrogen gas, hydrogen water

## Abstract

A simple titration (oxidimetry) method using a methylene blue-platinum colloid reagent is effective in determining the concentration of hydrogen gas in an aqueous solution. The method performs as effectively as the more complex and expensive electrochemical method.

## Background

Molecular hydrogen is useful for various novel medical and therapeutic applications. In air, hydrogen gas is potentially explosive, whereas, in an aqueous solution, it is safe and convenient to use. Recent biomedical studies have shown that hydrogen is a physiological regulatory factor that has antioxidant, anti-inflammatory, and antiapoptotic protective effects on cells and organs [1-5]. As a result, several aqueous solutions of hydrogen have been developed for use in medical applications as well as in health drinks.

A method for determining the concentration of hydrogen in water is very desirable, particularly if it is simpler and more inexpensive than the current state-of-the-art method involving expensive electrochemical gas sensors. Accordingly, we investigated a simple oxidimetry method that involves a redox reaction of methylene blue (MB) oxidant in the presence of a colloidal platinum (Pt) catalyst. MB is well known to react with an equimolar amount of hydrogen in the presence of Pt or palladium to produce colorless reduced MB (leucomethylene blue, leucoMB), as follows:

$$\text{MB }\left(\text{blue}\right)+\text{2}{\text{H}}^{+}+\text{2}{\text{e}}^{-}\to \text{leucoMB }\left(\text{colorless}\right)$$

**Figure 1:**
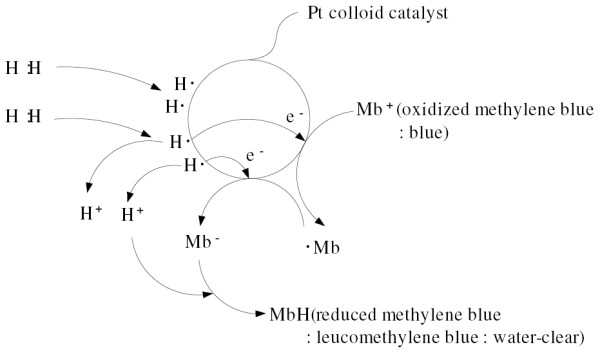
**Conceptual illustration of the reaction between H_2 _and MB-Pt**. 1 mole of hydrogen molecules reacts with 1 mole of methylene blue (MB) molecules to give 1 mole of reduced MB (leucomethylene blue, leucoMB) molecules.

On the basis of the above equation, we used a volumetric analysis method called oxidimetry to determine the concentration of hydrogen in water by a titration by using the MB-Pt reagent (Figure 1).

## Methods/Design

### Preparation of MB-Pt reagent

MB (0.3 g) (Waldeck-Gmbh & Co KG, Munster, Germany) was dissolved in 98% ethanol (98.9 g) to give a solution of MB (99.2 g) in ethanol. An aqueous suspension of 2% colloidal Pt (0.8 g) (Tanaka Kikinzoku Group Company) was added to the solution and the mixture was stirred to give 100 g of MB-Pt reagent (MiZ Company, Kanagawa, Japan). The reagent was distributed in small plastic bottles, from each of which one drop of the reagent (17 mg or 0.02 mL) was drawn.

### Preparation of hydrogen-rich water samples

Hydrogen-saturated water (0.8 mM) was prepared by bubbling hydrogen gas through purified water. Three concentrations of hydrogen-rich water (0.3, 0.2, and 0.1 mM) were prepared by diluting hydrogen-saturated water with purified water.

### Determination of hydrogen concentrations

Electrochemical determination of the hydrogen concentration was performed using an electrochemical gas sensor (model DHD1-1, DKK-TOA Corporation, Tokyo, Japan).

**Figure 2:**
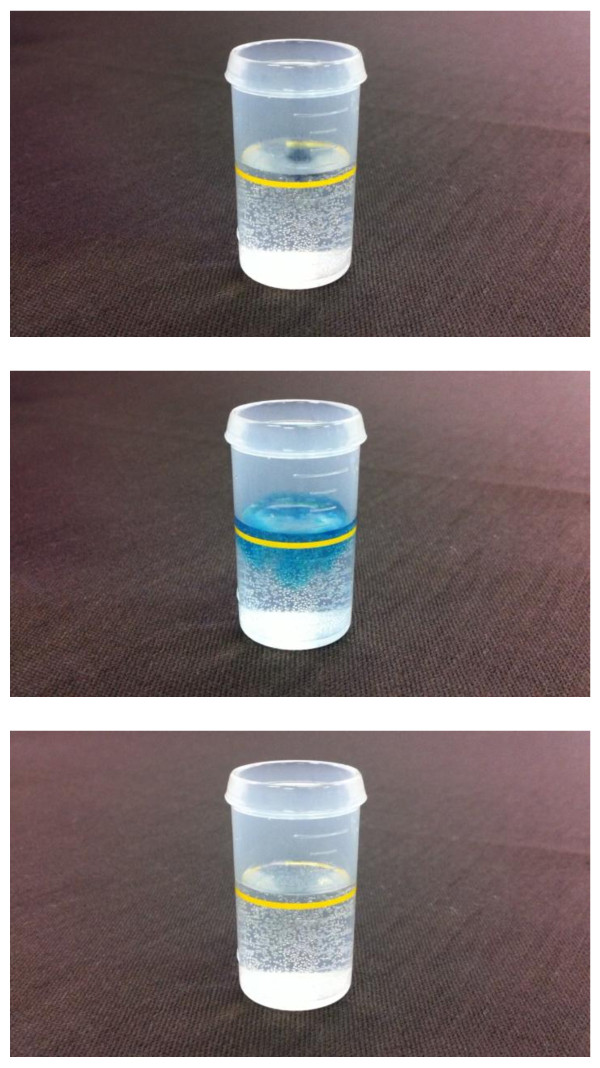
**H_2 _reduces MB (blue) to leucoMB (colorless)**. Early during the titration (MB-Pt reagent dropped into hydrogen-rich water), the solution contains more hydrogen than MB. Hence, MB is reduced to leucoMB and the solution is colorless. At the titration endpoint, the solution contains more MB than hydrogen; hence, it turns blue. 1. Drop an MB-Pt into hydrogen-rich water. 2. Hydrogen-rich water is colored by the blue of MB-Pt. 3. Immediately, blue turns colorless by hydrogen.

Oxidimetry determination of the hydrogen concentration was performed by a redox titration. The MB-Pt reagent was added dropwise to 20-mL samples of hydrogen-rich water until the solution changed from blue to colorless (Figure 2).

## Results/Discussion

One drop of the MB-Pt reagent provides 17 mg of the reagent. When the reagent is added to a hydrogen-rich water sample, the molar concentration of hydrogen in a sample is determined as follows:

$$\text{Moles of }{\text{H}}_{\text{2}}\text{ per sample }=0.017\times \left(0.3/100\right)/319.85\times \left(\text{number of drops consumed by titration}\right)$$

From this relationship, we infer that one drop (17 mg) of the reagent contains 0.16 μmol of MB. If the MB-Pt reagent is added dropwise to 20 mL of hydrogen-saturated water (0.8 mmol/L), approximately 100 drops of the reagent are required to reach the titration endpoint (when the solution turns from blue to colorless), as follows:

$$\begin{array}{c} 0.8\text{ mmol}/\left(1000\text{ mL}/20\text{ mL}\right)=16\;\mu \text{ mol}\\ 16\mu \text{mol}/0.16\;\mu \text{mol}/\text{drop }=100\text{ drops} \end{array}$$

However, when we added the MB-Pt reagent to 20 mL of hydrogen water (0.8 mmol/L), only 55 drops of the reagent were required to reach the titration endpoint. The difference between the calculated and actual values is attributed to the evaporation of hydrogen during the measurement time and the quantity difference between MB and hydrogen in water. Thus, we estimate that one drop of the MB-Pt reagent in 20 mL of hydrogen water is actually reduced by 0.29 μmol of hydrogen, as follows:

16μmol/55 drops =0.29μmol/drop

In other words, if 20 ml of hydrogen water reduces one drop of the MB-Pt reagent, the concentration of dissolved hydrogen (DH) is 14.5 μmol/L or 0.03 mg/L, as follows:

$$0.29\;\mu \text{mol}/\text{drop }\times \left(1000\text{ mL}/20\text{ mL}\right)=14.5\;\mu \text{mol}/\text{Lor }0.03\text{ mg}/\text{L}$$

Similarly, if 20 ml of hydrogen water reduces three drops of the MB-Pt reagent, the concentration of DH is 43.6 μmol/L or 0.09 mg/L.

Table 1 lists the DH concentrations determined by both the known electrochemical method and our oxidimetry method. The two methods yield approximately equivalent results.

**Table 1:** Concentrations of Dissolved Hydrogen in Hydrogen Water, Determined by Electrochemical and Oxidimetry Methods

HW	DH (mg/l)	MB-Pt	MB→DH (mg/l)	DO (mg/l)	T (centigrade)
0.8 mM	1.60	55	1.65	0.54	24.8
	1.60	55	1.65	0.54	24.8
	1.62	56	1.68	0.45	23.7
	1.58	54	1.62	0.43	23.8

0.3 mM	0.62	19	0.57	1.6	25.3
	0.62	20	0.6	1.6	25.3
	0.60	18	0.54	2.1	23.5
	0.62	19	0.57	2.3	23.8

0.2 mM	0.41	12	0.36	2.1	25.1
	0.42	13	0.39	2.3	25.1
	0.41	13	0.39	2.4	23.6
	0.39	12	0.36	2.4	23.7

0.1 mM	0.22	5	0.15	3.0	24.8
	0.22	5	0.15	3.4	24.8
	0.20	4	0.12	4.3	23.8
	0.19	4	0.12	4.4	23.7

HW: hydrogen water, DH: dissolved hydrogen, MB-Pt: number of drops of methylene blue with colloidal platinum at the titration endpoint, MB→DH: calculated value of DH from MB-Pt, DO: dissolved oxygen, T: water temperature. DO was measured by a DO meter (model D-25 DO meter, Horiba).

Although DO was degassed through the process of hydrogen bubbling for hydrogen-saturated water (0.8 mmol/L), DO in purified water used for dilution was mixed with hydrogen-saturated water, which caused an increase in DO in hydrogen water with dilution magnification. This seemed to cause a deviation in the DH values obtained using the oxidimetry method from those using electrochemical method at a lower concentration of DH.

To study the influence of DO in hydrogen-rich water, we also measured DO-regulated hydrogen water prepared by diluting hydrogen-saturated water with DO-regulated purified water, where DO was degassed by bubbling inert N_2 _gas through purified water (Table 2).

**Table 2:** Concentrations of Dissolved Hydrogen in DO-Regulated Hydrogen water, Determined by Electrochemical and Oxidimetry Methods

HW	DH (mg/l)	MB	MB→DH (mg/l)	DO (mg/l)	T (centigrade)
0.8 mM	1.60	55	1.65	0.54	24.8
	1.60	55	1.65	0.54	24.8
	1.62	56	1.68	0.45	23.7
	1.58	54	1.62	0.43	23.8

0.3 mM	0.61	21	0.63	0.62	23.7
	0.61	21	0.63	0.62	23.7
	0.61	21	0.63	0.87	25.2
	0.62	20	0.6	0.88	25.3

0.2 mM	0.41	14	0.42	0.68	23.8
	0.41	13	0.39	0.68	23.7
	0.40	13	0.39	0.89	25.1
	0.41	13	0.39	0.87	25.1

0.1 mM	0.19	6	0.18	0.81	23.5
	0.19	6	0.18	0.81	23.5
	0.20	6	0.18	0.88	25.1
	0.19	6	0.18	0.91	25.1

The values of hydrogen water for 0.8 mM are quoted from Table 1.

The DH values obtained from the oxidimetry method approached those obtained using the electrochemical method. DO as an oxidant would compete with MB when molecular hydrogen reduces MB on the surface of Pt.

### •Statistical analysis

Sixteen respective observed values obtained using the electrochemical and the oxidimetry methods were analyzed, premising that these values correspond to each another.

A linear relationship between the values obtained using the oxidimetry method and those obtained using the electrochemical method was shown. This relationship was found using a regression model (linear regression model) having regression coefficients, in which the values obtained using the oxidimetry method were regarded as response variables and those obtained using the electrochemical method were regarded as explanatory variables. The amount of information that can be explained by the straight line was indicated by the coefficient of determination (R^2^).

Moreover, a second-order component was added to the regression model to determine whether it deviates from linearity. In addition, the influence of DO and water temperature on the oxidimetry method was also determined.

### •Analysis results

#### 1. Linear relation equation

**Figure 3:**
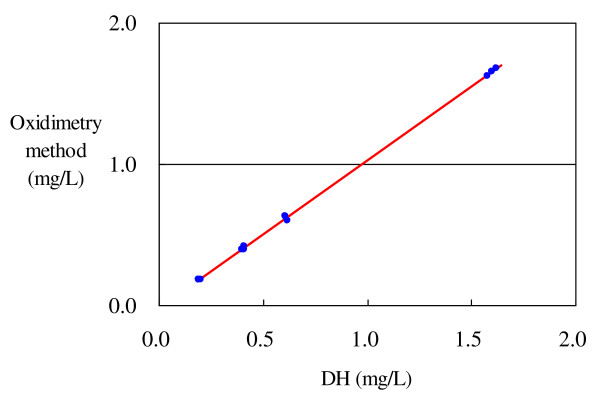
**Oxidimetry method and electrochemical methods for DO-regulated hydrogen water**. Value obtained by the oxidimetry method = -0.0229 + 1.0459 × value obtained by the electrochemical method.

The linear regression equation for the electrochemical and the oxidimetry methods for DO-regulated hydrogen water is given below. Here, the correlation coefficient r was 0.9998, the coefficient of determination R^2 ^was 0.9995, suggesting that the linear line indicated 99.95% of the information, and the deviation from the linear line or the standard deviation (error standard deviation) was 0.0129 (Table 3 and Figure 3).

**Table 3:** Linear regression equation for the electrochemical and the oxidimetry methods for DO-regulated hydrogen water

Parameter	Regression coefficient	Standard error	t value	p value
Intercept	-0.0229	0.0053	-4.33	0.0007
Electrochemical method	1.0459	0.0060	175.38	< 0.0001
		
Coefficient of determination	Correlation coefficient	Error standard deviation		
		
0.9995	0.9998	0.0129		

Valueobtained bythe oxidimetry method=-0.0229+1.0459×value obtained by the electrochemical method

**Figure 4:**
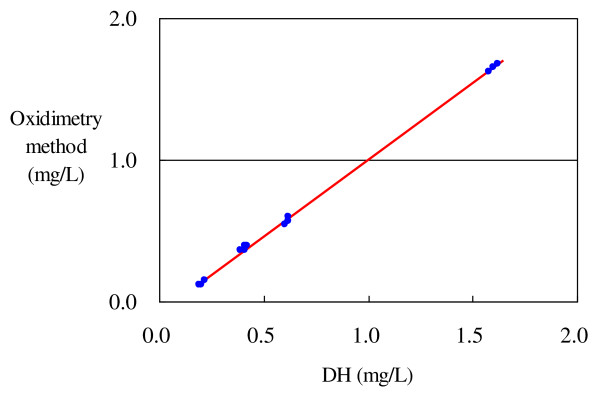
**Oxidimetry and electrochemical methods for DO-unregulated hydrogen water**. Value obtained by the oxidimetry method = -0.0837 + 1.0829 × value obtained by the electrochemical method.

The linear regression equation for DO-unregulated hydrogen water is given below. Here, the correlation coefficient r was 0.9997, the coefficient of determination R^2 ^was 0.9993, suggesting that the linear line indicated 99.93% of the information, and the deviation from the linear line or the standard deviation (error standard deviation) was 0.0158 (Table 4 and Figure 4).

**Table 4:** Linear regression equation for the electrochemical and the oxidimetry methods for DO-unregulated hydrogen water

Parameter	Regression coefficient	Standard error	t value	p value
Intercept	-0.0837	0.0066	-12.75	< 0.0001
Electrochemical method	1.0829	0.0074	146.47	< 0.0001
		
Coefficient of determination	Correlation coefficient	Error standard deviation		
		
0.9993	0.9997	0.0158		

Valueobtained bytheoxidimetry method=-0.0837+1.0829×valueobtained bythe electrochemical method

#### 2. Examination of linearity

To examine linearity between the electrochemical and the oxidimetry methods, a second-order term b2 was added to the linear regression model as 'y = b0 + b1 × × + b2 × x^2^', and the significance level was determined (where b2 is regarded as significant if there is curvature with a second- or a higher-order term).

As a result, the second-order components for DO-regulated hydrogen water (p = 0.8974) and DO-unregulated hydrogen water (p = 0.7429) were not statistically significant, and the relationship between the electrochemical and the oxidimetry methods was linear in both cases (Tables 5 and 6, respectively).

**Table 5:** Examination of curvature in the electrochemical and the oxidimetry methods for DO-regulated hydrogen water

Parameter	Regression coefficient	Standard error	t value	p value
Intercept	-0.0243	0.0120	-2.02	0.0647
Electrochemical method: first-order component	1.0508	0.0377	27.84	< 0.0001
Electrochemical method: second-order component	-0.0026	0.0197	-0.13	0.8974

**Table 6:** Examination of curvature in the electrochemical and the oxidimetry methods for DO-unregulated hydrogen water

Parameter	Regression coefficient	Standard error	t value	p value
Intercept	-0.0790	0.0156	-5.07	0.0002
Electrochemical method: first-order component	1.0669	0.0483	22.08	< 0.0001
Electrochemical method: second-order component	0.0084	0.0251	0.34	0.7429

#### 3. Influence of DO and water temperature on the oxidimetry method

The influence of DO and water temperature on the oxidimetry method was studied using a multiple regression model having values measured by the oxidimetry method as response variables and the values measured by the electrochemical method, values measured by the DO meter (mg/L), water temperature (degree Celsius), and the interaction between the electrochemical method and DO as explanatory variables. The result showed that water temperature is not statistically significant (Table 7).

**Table 7:** Statistical test results of the multiple regression model having values measured by the oxidimetry method as response variables, values measured by the electrochemical method, values measured by the DO meter (mg/L), water temperature (degree Celsius), and the interaction between the electrochemical method and DO as explanatory variables

		Type III			
Source	Degree of freedom	Sum of squares	Mean sum of squares	F ratio	p value
Electrochemical method	1	2.5041	2.5041	12891.5	< 0.0001
DO meter (mg/L)	1	0.0018	0.0018	9.18	0.0060
Water temperature (degree Celsius)	1	0.0003	0.0003	1.67	0.2088
Interaction between electrochemical method and DO	1	0.0014	0.0014	7.46	0.0119

The influence of only DO on the oxidimetry method was then studied using a multiple regression model with the same response and explanatory variables mentioned above, except for water temperature.

The relationship between the oxidimetry method, electrochemical method, DO, and the interaction between electrochemical method and DO is given below. As DO increases by 1.0, the value obtained by the oxidimetry method drops 0.0117, and the gradient for the oxidimetry and the electrochemical methods drops 0.0358 from +1.0575 (Table 8).

**Table 8:** Relationship among the oxidimetry method, the electrochemical method, and DO for DO-regulated hydrogen water (multiple regression analysis)

Parameter	Regression coefficient	Standard error	t value	p value
Intercept	-0.0076	0.0074	-1.02	0.316
Electrochemical method	1.0575	0.0094	112.96	< 0.0001
DO meter (mg/L)	-0.0117	0.0041	-2.82	0.0094
Interaction between electrochemical method and DO	-0.0358	0.0126	-2.85	0.0088

$$\begin{array}{c} \text{Value}\;\text{obtained by}\;\text{the}\;\text{oxidimetry method}=-0.0076+1.0575\times \text{value}\;\text{obtained by}\;\text{theelectrochemical method }-0.0117\\ \times \;\text{DO}-0.0358\times \text{interaction between electrochemical method and DO} \end{array}$$

## •Discussion

The linear relationship between the electrochemical and the oxidimetry methods for DO-regulated hydrogen water is obtained as shown below. Here, the intercept is almost 0 (-0.0229), and the gradient of the line is 1.0459 or approximately 1. These findings indicate that the oxidimetry method reflects the numerical values obtained using the electrochemical method correctly, i.e., the accuracy is sufficient.

Valueobtained bytheoxidimetry method=-0.0229+1.0459×valueobtained bytheelectrochemical method

The correlation coefficient r is 0.9998, the coefficient of determination R^2 ^is 0.9995, suggesting that the linear line indicates 99.95% of the information, and the deviation from the linear line or the standard deviation is 0.0129, which is almost equal to the minimum displaying unit of 0.01. The results show that the oxidimetry method has sufficient precision (accuracy) and can be used as a substitute for the electrochemical method.

The linear regression equation for DO-unregulated hydrogen water is given below.

Valueobtained bytheoxidimetry method =-0.0837+1.0829×valueobtained bytheelectrochemical method

Multiple regression analysis reveals that as DO increases by 1.0, the value based on the oxidimetry method drops 0.0117, and the gradient of the oxidimetry method and the electrochemical method drops 0.0358.

The measured hydrogen concentration of the solution was approximately between 0.2 and 1.6 mg/L at this time. The oxidimetry method was practically sufficient to measure the hydrogen concentration to one decimal place within this range. The oxidimetry method is inferred to be useful for a substitute for the electrochemical method.

## List of abbreviations

DH: dissolved hydrogen; DO: dissolved oxygen; H_2_: molecular hydrogen; MB: methylene blue; MB-Pt: methylene blue-platinum colloid; Pt: platinum.

## Competing interests

This study protocol was funded by MiZ Company. Tomoki Seo, Ryosuke Kurokawa and Bunpei Sato are all employees of MiZ Company.

## Authors' contributions

The authors equally contributed to the production of this article and have read and approved the final manuscript.
